# The Efficacy of Lacosamide in the Treatment of Delirium Complicated With Ileus: Four Cases

**DOI:** 10.1002/npr2.70102

**Published:** 2026-02-20

**Authors:** Shinji Sato

**Affiliations:** ^1^ Department of Psychiatry, Ibaraki Clinical Educational and Training Center, Faculty of Medicine University of Tsukuba Tsukuba Japan; ^2^ Department of Psychiatry Ibaraki Prefectural Hospital Kasama Japan

**Keywords:** delirium, ileus, lacosamide, medication

## Abstract

Delirium is a common disease in elderly patients and requires medications, although nondrug interventions are recommended. When delirious patients are complicated with ileus, the selection of drugs would be difficult. In this paper, three cases of antipsychotic‐induced paralytic ileus successfully managed with lacosamide are presented without any severe adverse effects, including psychiatric symptoms. Lacosamide is a newer antiepileptic drug that acts on the sodium channels. Drug‐induced eruptions are rare compared to other antiepileptics, such as carbamazepine and lamotrigine. Lacosamide can be administered intravenously as well as orally. The mechanisms of ileus have been suggested; to involve antagonism of the cholinergic, histaminergic, and serotonergic systems. Since lacosamide is not related to these antagonists, it may be one of the helpful options for treating delirium tremens in elderly patients.

## Abstract 2

Delirium in elderly patients is difficult to treat when complicated by ileus. This report describes three cases of antipsychotic‐induced paralytic ileus successfully managed with lacosamide without severe adverse effects. Lacosamide's pharmacological profile suggests it may be a safe treatment option for delirium tremens in older patients.

## Graphical abstract

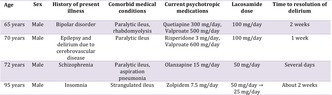

## 1 Introduction

Though nondrug interventions are preferred in the treatment of delirium, in actual clinical practice, medication is often needed. When patients suffer from delirium complicated by ileus or aspiration pneumonia, to the best of my knowledge, appropriate interventions for this condition have not been reported in the literature. This paper presents three cases of antipsychotic‐induced paralytic ileus successfully treated with lacosamide without the occurrence of serious adverse effects. Biassoni et al. [1] presented a case report demonstrating the efficacy of lacosamide for epileptic seizures, with concomitant improvement in delirium. However, there are few reports suggesting that lacosamide improves delirium. Thus, this case report may be considered clinically valuable. Informed consent was obtained from all patients, and modifications to the data were made to ensure anonymity.

## 2 Case Presentation

Mr. A, a 65‐year‐old male, was an outpatient of X psychiatric clinic and was diagnosed with bipolar disorder. He complained of abdominal pain and was taken to Y hospital. After examination, he was diagnosed with paralytic ileus and was admitted directly to Y hospital. He was taking 300 mg/day of quetiapine and 500 mg/day of valproate. After admission, his medication was immediately discontinued, and his food intake was also stopped. On the first night of hospitalization, he experienced insomnia, agitation, and visual hallucinations. He was diagnosed with delirium. In the case of food intake cessation, it might be possible to use the blonanserin patch to improve delirium. However, his laboratory data indicated that serum CK was very high, at over 2000 U/L. This might occur due rhabdomyolysis and so the use of the patch, which could move his condition to malignant state, should be avoided. For this reason, he was started on a regimen of 50 mg of intravenous lacosamide with 50 cc of saline solution, twice a day. His nocturnal restlessness subsided, and the drug was continued until the ileus resolved, about two weeks after hospitalization. No adverse events were observed.

Mr. B, a 70‐year‐old male, had a history of seizures and cerebral hemorrhages in the right side of his brain due to cerebrovascular disease. He was taking 600 mg of valproate and 3 mg of risperidone per day because he tended to be agitated and excited. He was admitted to Y hospital and diagnosed with paralytic ileus caused by antipsychotics. His medications were discontinued, and oral intake was withheld. Three days after admission, he became delirious and required treatment. However, the use of antipsychotics could lower the threshold for seizures. Therefore, he was started on 100 mg of intravenous lacosamide twice a day. One week later, his delirium improved, and after his ileus improved, he resumed taking his medication and was discharged from the hospital.

Mr. C, a 72‐year‐old male with schizophrenia, was an outpatient of Z psychiatric hospital. His mental status was stable with the administration of 15 mg of olanzapine per day. He was admitted to Y Hospital with acute abdominal pain. He was diagnosed with drug‐induced paralytic ileus and aspiration pneumonia. After admission, his medication and oral intake were discontinued. The next day, he began experiencing delirium. Dopamine antagonists can cause difficulty in swallowing and also extrapyramidal symptoms that reduce swallowing function by reducing substance P [2]. Therefore, instead of using antipsychotics or dopamine antagonists, he was started on an infusion of 50 mg of lacosamide administered in two divided doses in the evening and before sleeping. The delirium soon improved, and after the ileus and pneumonia improved, the patient was transferred back to Z psychiatric hospital.

Mr. D was a 95‐year‐old male with no prior psychiatric history. Before admission, he had been taking zolpidem 7.5 mg for insomnia. He was admitted to Z Hospital with a strangulated ileus. On the eighth day of hospitalization, nocturnal agitation occurred. Because oral intake was prohibited, oral medications could not be administered, and blonanserin patches were initiated. Despite titrating up to 40 mg of blonanserin patches, the delirium did not improve, and hypotension was observed. Subsequently, intravenous lacosamide was subsequently initiated at a dose of 50 mg per day, administered in two divided doses in the evening and at night. However, he became over‐sedated. Thus, the lacosamide dose was reduced to 25 mg per day. From the next day, the sedation resolved; furthermore, nocturnal agitation disappeared and sleep was maintained. On hospital day 12, oral intake was resumed, and he continued taking lacosamide at the same dose via the oral route. Three weeks after admission, both the delirium and strangulated ileus had improved, and he was discharged.

## 3 Discussion

All four cases were diagnosed with ileus, thus preventing the use of antipsychotics with anticholinergic effects. In addition, the presence of rhabdomyolysis, epilepsy, and aspiration pneumonia made it necessary to avoid administering dopamine antagonist drugs. To date, no treatments for delirium have been approved by the Food and Drug Administration. Thus, antipsychotics or dexmedetomidine are commonly selected for its management [3]. However, these interventions have notable limitations. Antipsychotic use may cause extrapyramidal symptoms, prolongation of the baseline QTc interval, or anticholinergic side effects, whereas dexmedetomidine may be beneficial but can induce excessive sedation or hypotension and require close hemodynamic monitoring and intensive care resources, particularly in elderly patients [3]. Furthermore, there appears to be no available literature on the pharmacological management of delirium complicated by the aforementioned conditions, including the use of not only antipsychotics but also anticonvulsant agents. In addition, because oral intake was impossible, it was necessary to administer sedative medications intravenously to treat their delirium.

Lacosamide, like lamotrigine or carbamazepine, is an antiepileptic drug that acts on the sodium channels [4]. However, unlike these agents, drug eruptions are rare compared to other anticonvulsants [4]. Lacosamide can be administered intravenously as well as orally. The most common adverse effects of lacosamide are dizziness, headache, nausea, and vomiting. The half‐life is 14–16 h [4]. Some reviews reported that the effects of lacosamide on mental status and cognitive function were almost equal to placebo, and lacosamide improved psychiatric symptoms in epilepsy patients with depression, mania, and anxiety [5, 6]. Bianco et al. reported favorable efficacy and tolerability of lacosamide at low doses in elderly patients with epilepsy [7]. Although the incidence of adverse effects is relatively low, the most commonly reported events include dizziness, fatigue, and visual disturbances [8]. Furthermore, Kubo et al. [9] found that while antiepileptic drugs overall were associated with an increased risk of delirium in patients aged ≥ 70 years (OR, 1.82; 95% CI, 1.47–2.27), lacosamide was not associated with a significantly higher risk compared with other antiepileptic agents (OR, 1.92; 95% CI, 0.72–5.08). However, previous studies, including a pharmacovigilance analysis of the FAERS database by Yang et al. [10], have suggested that lacosamide may cause rare but clinically relevant cardiac adverse events, including mild PR interval prolongation and atrioventricular block, particularly in elderly patients.

Though the occurrence of delirium has been shown to be associated with electrolyte abnormalities, few studies have addressed the role of sodium channels. Eijkelkamp et al. [11] suggested that the activity of voltage‐gated sodium channels may be implicated in various brain dysfunctions, including epilepsy, chronic pain and autistic spectrum disorders. In particular, in the pathophysiology of delirium, Sher et al. [3]. pointed out that delirium is associated with multiple mechanisms, including neuronal aging, oxygen deprivation, neuroinflammation, oxidative and physiological stress, and neurotransmitter imbalances involving the cholinergic, dopaminergic, serotonergic, GABAergic, histaminergic, and melatonergic systems. They suggested that inhibition of the activity of voltage‐gated sodium channels, which is the mechanism of lacosamide, may decrease neuronal excitability [3]. This mechanism may be associated with protection against NMDA receptor‐mediated neurotoxicity, which could be relevant to delirium improvement [3].

The mechanisms of constipation or ileus remain unclear, but it has been suggested that antagonism of not only the cholinergic system but also the histaminergic and serotonergic systems would be involved [12]. Therefore, lacosamide, which is not related to these antagonists, may be appropriate in cases of ileus.

In this report, the dosage of lacosamide varied among cases. In the absence of an established protocol for the use of lacosamide in delirious patients, dose selection was guided by individual factors, including age, physical conditions, and severity of delirium. In each case, lacosamide was initiated cautiously and adjusted according to clinical response, with careful monitoring for adverse effects.

## 4 Conclusion

Lacosamide might be considered one of the options for intervening in elderly patients with delirium tremens who are unable to take oral medications because of medical comorbidities and in whom avoidance of severe adverse effects is particularly important. However, this report included only four patients, which inevitably limits the generalizability of the findings. Further larger‐scale investigations, including randomized controlled trials, are required to more rigorously evaluate the efficacy and safety of lacosamide for delirium complicated by ileus.

## Author Contributions

Shinji Sato is the only author of this manuscript.

## Funding

The author has nothing to report.

## Consent

Written informed consents were obtained from all patients.

## Conflicts of Interest

The author declares no conflicts of interest.

## Data Availability

All data supporting the findings of this case report are included in the text.

## References

[1] C. D. Bianco , F. Placidi , C. Liguori , et al., “Long‐Term Efficacy and Safety of Lacosamide and Levetiracetam Monotherapy in Elderly Patients With Focal Epilepsy: A Retrospective Study,” Epilepsy & Behavior 94 (2019): 178–182, 10.1016/j.yebeh.2019.02.022. 30959275

[2] T. Nagamine , “Serum Substance P Levels in Patients With Chronic Schizophrenia Treated With Typical or Atypical Antipsychotics,” Neuropsychiatric Disease and Treatment 4 (2008): 289–294, 10.2147/ndt.s2367. 18728797 PMC2515891

[3] Y. Sher , A. C. M. Cramer , and A. Ament , “Acid for Treatment of Hyperactive or Mixed Delirium: Rationale and Literature Review,” Psychosomatics 56 (2015): 615–625, 10.1016/j.psym.2015.09.008. 26674479

[4] W. Cawello , “Clinical Pharmacokinetic and Pharmacodynamic Profile of Lacosamide,” Clinical Pharmacokinetics 54 (2015): 901–914. 25957198 10.1007/s40262-015-0276-0

[5] I. Cuomo , D. Piacentino , and G. D. Kotzalidis , “Lacasamido in Bipolar Disorders: A 30‐Day Comparison to a Retrospective Control Group Treated With Other Antiepileptics,” Psychiatry and Clinical Neurosciences 72, no. 12 (2018): 864–875, 10.1111/pcn.12784. 30251375

[6] R. Rocamora , M. Ley , A. Molins , et al., “Effect of Lacosamide o Depression and Anxiety Symptoms in Patients With Focal Refractory Epilepsy: A Prospective Multicenter Study,” Epilepsy & Behavior 79 (2018): 87–92. 29253680 10.1016/j.yebeh.2017.10.032

[7] E. Biassoni , M. Bellucci , E. Micalizzi , et al., “The Role of EEG and Neuroimaging in the Diagnosis of Non‐Convulsive Status Epilepticus in Subacute Encephalopathy With Seizures in Alcoholics (SESA Syndrome): A Case Report and Overview of the Literature,” Neurological Sciences 45, no. 10 (2024): 5053–5062, 10.1007/s10072-024-07609-2. 38802690

[8] C. Yang , Y. Peng , L. Zhang , and L. Zhao , “Safety and Tolerability of Lacosamide in Patients With Epilepsy: A Systematic Review and Meta‐Analysis,” Frontiers in Pharmacology 12 (2021): 694381, 10.3389/fphar.2021.694381. 34616294 PMC8488108

[9] T. Kubo , R. Sogawa , S. Tsuruhashi , et al., “Risk of Delirium With Antiepileptic Drug Use: A Study Based on the Japanese Adverse Drug Event Report Database,” International Journal of Clinical Pharmacy 45 (2023): 1260–1266. 36977859 10.1007/s11096-023-01564-2

[10] C. Yang , W. Zhao , H. Chen , Y. Yao , and J. Zhang , “Cardiac Adverse Events Associated With Lacosamide: A Disproportionality Analysis of the FAERS Database,” Scientific Reports 14 (2024): 16202, 10.1038/s41598-024-67209-0. 39003359 PMC11246456

[11] N. Eijkelkamp , J. E. Linley , and M. D. Mark D Baker , “Neurological Perspectives on Voltage‐Gated Sodium Channels,” Brain 135, no. 9 (2012): 2585–2612, 10.1093/brain/aws225. 22961543 PMC3437034

[12] Y. Xu , N. Amdanee , and X. Zhang , “Anti‐Psychotic‐Induced Constipation: A Review of the Pathogenesis, Clinical Diagnosis, and Treatment,” CNS Drugs 35 (2021): 1265–1274. 34427901 10.1007/s40263-021-00859-0

